# Engineered allele substitution at *PPARGC1A* rs8192678 alters human white adipocyte differentiation, lipogenesis, and PGC-1α content and turnover

**DOI:** 10.1007/s00125-023-05915-6

**Published:** 2023-05-12

**Authors:** Mi Huang, Melina Claussnitzer, Alham Saadat, Daniel E. Coral, Sebastian Kalamajski, Paul W. Franks

**Affiliations:** 1 Department of Clinical Sciences, Genetic and Molecular Epidemiology Unit, Clinical Research Centre, Lund University, Malmö, Sweden; 2 Metabolism Program, Broad Institute of MIT and Harvard, Cambridge, MA 02142 USA; 3 Department of Nutrition, Harvard T.H. Chan School of Public Health, Boston, MA USA

**Keywords:** Gene and environment interaction, GWAS, Obesity, PGC-1α, *PPARGC1A*, rs8192678

## Abstract

**Aims/hypothesis:**

*PPARGC1A* encodes peroxisome proliferator-activated receptor γ coactivator 1-α (PGC-1α), a central regulator of energy metabolism and mitochondrial function. A common polymorphism in *PPARGC1A* (rs8192678, C/T, Gly482Ser) has been associated with obesity and related metabolic disorders, but no published functional studies have investigated direct allele-specific effects in adipocyte biology. We examined whether rs8192678 is a causal variant and reveal its biological function in human white adipose cells.

**Methods:**

We used CRISPR-Cas9 genome editing to perform an allelic switch (C-to-T or T-to-C) at rs8192678 in an isogenic human pre-adipocyte white adipose tissue (hWAs) cell line. Allele-edited single-cell clones were expanded and screened to obtain homozygous T/T (Ser482Ser), C/C (Gly482Gly) and heterozygous C/T (Gly482Ser) isogenic cell populations, followed by functional studies of the allele-dependent effects on white adipocyte differentiation and mitochondrial function.

**Results:**

After differentiation, the C/C adipocytes were visibly less BODIPY-positive than T/T and C/T adipocytes, and had significantly lower triacylglycerol content. The C allele presented a dose-dependent lowering effect on lipogenesis, as well as lower expression of genes critical for adipogenesis, lipid catabolism, lipogenesis and lipolysis. Moreover, C/C adipocytes had decreased oxygen consumption rate (OCR) at basal and maximal respiration, and lower ATP-linked OCR. We determined that these effects were a consequence of a C-allele-driven dysregulation of PGC-1α protein content, turnover rate and transcriptional coactivator activity.

**Conclusions/interpretation:**

Our data show allele-specific causal effects of the rs8192678 variant on adipogenic differentiation. The C allele confers lower levels of *PPARGC1A* mRNA and PGC-1α protein, as well as disrupted dynamics of PGC-1α turnover and activity, with downstream effects on cellular differentiation and mitochondrial function. Our study provides the first experimentally deduced insights on the effects of rs8192678 on adipocyte phenotype.

**Graphical Abstract:**

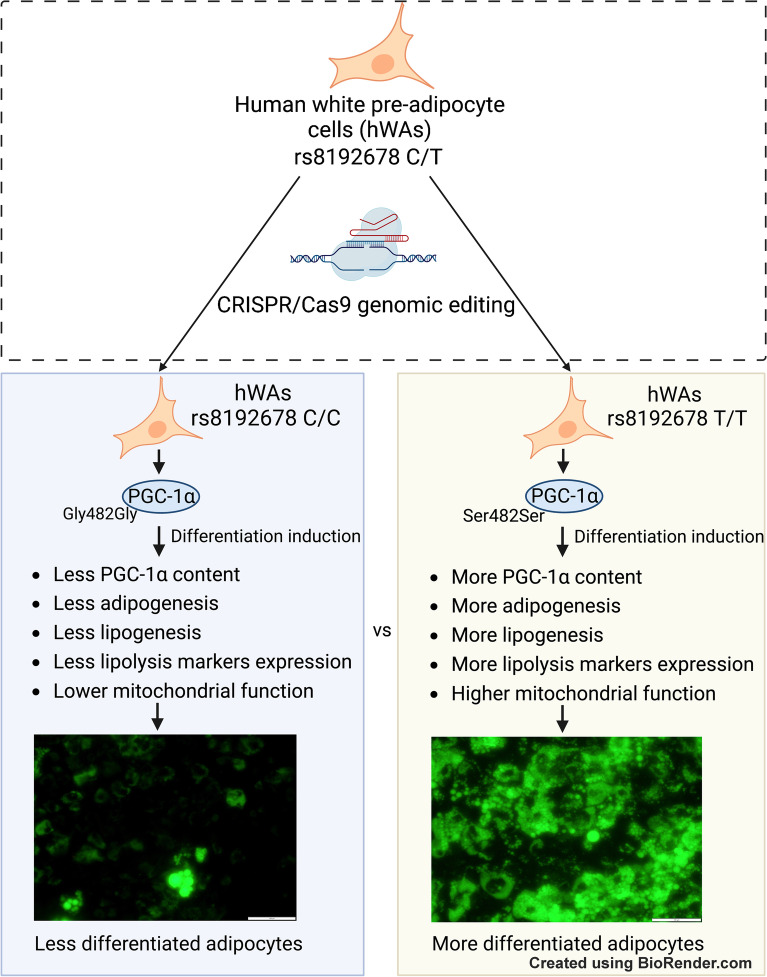

**Supplementary Information:**

The online version of this article (10.1007/s00125-023-05915-6) contains peer-reviewed but unedited supplementary material.

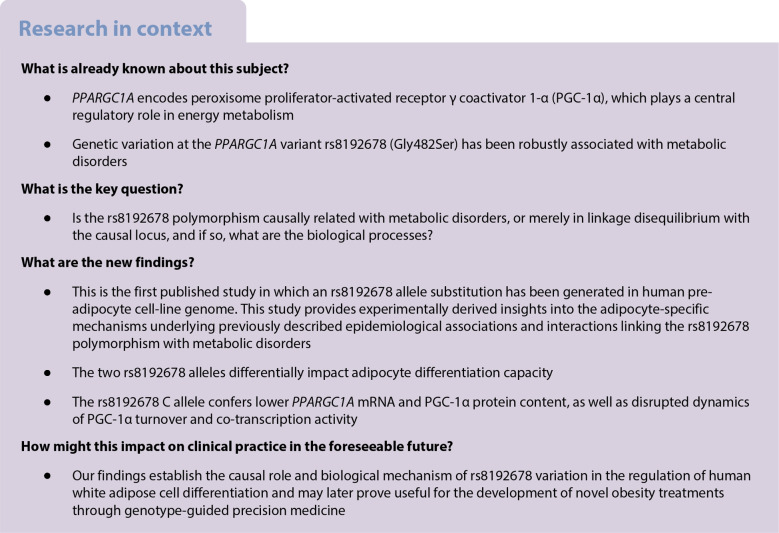



## Introduction

Globally, more than 650 million adults were estimated to be obese in 2016, as reported by the WHO [1]. Obesity, causing millions of deaths annually, is also a major risk factor for type 2 diabetes, cardiovascular disease and certain cancers, amongst many other health conditions [2, 3]. Obesity is a consequence of complex interactions between genetic factors and lifestyle [4] and typically occurs when white adipose tissue (WAT) cells accumulate and store excess lipids.

Certain weight loss-promoting lifestyle interventions, including dietary restriction and exercise, promote lipolysis in WAT and thermogenesis in brown adipose tissue (BAT) [5]. Many genetic variants have been implicated in diet-induced weight loss, often through post hoc analysis of lifestyle intervention trials [6]. Experimental studies in animals and humans have identified *PPARGC1A* as a strong genetic mediator of these effects, although allele-specific functional studies are yet to be performed [7]. Genetic variation in *PPARGC1A* has also been examined in the context of weight loss trials, with the Gly482Ser variant (rs8192678, C/T alleles) emerging as one of the most robust signals [8].

*PPARGC1A* encodes peroxisome proliferator-activated receptor γ coactivator 1-α (PGC-1α), which plays a central regulatory role in energy metabolism [9] by modulating systemic oxidative metabolism and mitochondrial function [10]. The rs8192678 minor T allele frequency varies from 5% in African populations to 26–44% prevalence in American, East Asian, South Asian and European populations. The rs8192678 T allele has been linked with type 2 diabetes [11, 12], with risk odds ratios ranging from 1.2 to 5.2 in different ethnicities, and with insulin resistance (the data are summarised in another paper [8]). The same allele also associates with lower *PPARGC1A* mRNA expression in muscle and islets [13, 14]. With regard to protein turnover rate, the T allele-encoded 482Ser PGC-1α degrades faster than the C allele-encoded 482Gly protein when episomally overexpressed in HepG2 cells and Ins-1 cells [15, 16]. Collectively, these studies suggest that the rs8192678 polymorphism may affect PGC-1α protein abundance, which in turn may disrupt mitochondrial biogenesis and function. However, the biological mechanisms linking the Gly482Ser PGC-1α variant with obesity are poorly understood. Despite abundant epidemiological data on the association of rs8192678 with metabolic disorders, no studies have investigated the genomic allele-specific effects on cells with an isogenic genetic background. To address this, we performed CRISPR-Cas9-mediated allele editing of rs8192678 in human white pre-adipocyte cell lines, differentiated the cells to adipocytes, and studied their adipocyte-specific and mitochondrial phenotype, as well as investigated the effect of the Gly482Ser mutation on PGC-1α turnover and transcriptional activity.

## Methods

### Cell culture and differentiation

Immortalised human white pre-adipocyte tissue (hWAs) cells were obtained from Y.H. Tseng (Joslin Diabetes Center, Harvard Medical School, USA) [17]. Cells were maintained in high glucose DMEM medium with GlutaMAX (31966047, Thermo Fisher Scientific, Sweden) supplemented with 10% (vol./vol.) FBS (SV30160.03, HyClone, USA) and 1% (vol./vol.) (100 U/ml) penicillin/streptomycin (178-111291, Thermo Fisher Scientific) at 37ºC and 5% (vol./vol.) CO_2_. During the culturing process, mycoplasma contamination was regularly checked. To differentiate hWAs pre-adipocytes into mature adipocytes, the cells were seeded into 6-, 24- or 96-well plates with a density of 160,000, 40,000 or 8000 cells per well, respectively. Fully confluent cells were incubated in differentiation medium for at least 12 days, as described elsewhere [17]. The differentiation medium was replaced every 3 days. As the adipocytes were differentiated in 25 mmol/l glucose medium, the differentiated cells were adapted to 5.5 mmol/l glucose DMEM medium, to mimic physiological glucose conditions in vivo, for 3 days prior to performing functional assays.

### sgRNA and ssDNA design for CRISPR-Cas9-mediated single nucleotide editing of rs8192678

Prior to designing single guide RNA (sgRNA) and donor template, genomic DNA from hWAs cells was extracted using DNeasy Blood and Tissue kit (69506, Qiagen, Sweden) and used in PCR to amplify a 742 bp DNA fragment surrounding rs8192678. The sequences of the primers used were listed in electronic supplementary material (ESM) Table 1. Sanger sequencing was then used to confirm the DNA sequence of the amplicon. sgRNA and donor template were designed using Integrated DNA Technologies (IDT, CA, USA) custom design tool. As hWAs cells are heterozygous (C/T) at rs8192678, two sgRNAs and two donor templates were designed for substituting either C-to-T or T-to-C alleles in the genome, as listed in ESM Table 1. To generate rs8192678 heterozygous C/T cell clones, a pre-designed scrambled CRISPR RNA (crRNA; 1072544, IDT) was used. All oligonucleotides, sgRNAs and ssDNAs were purchased from IDT.

### Electroporation of hWAs cells

Electroporation for the delivery of the CRISPR-Cas9 toolkit has previously been successfully applied in pre-adipocytes [18, 19]. To achieve adequate CRISPR editing efficiency, we used electroporation to deliver complexed sgRNA and Cas9 protein (IDT) into the cells. Briefly, 150 pmol sgRNA and 150 pmol Cas9 protein were incubated for 20 min to form ribonucleoprotein (RNP) complex, then mixed with 2×10^5^ cells resuspended in 100 μl nucleofector reagent L (VCA-1005, Lonza, Sweden). The electroporation reaction was performed immediately using the program A-033 on a Nucleofector 2b device (Lonza). The transfected cells were transferred into a 6-well plate containing antibiotic-free growth medium supplemented with 30 μmol/l homology-dependent repair (HDR) enhancer (1081072, IDT) cultured for 2 days at 32ºC, then transferred to 37ºC.

### Clone selection and genotyping

Upon reaching 80% confluence, the cells were split for further expansion or for DNA extraction to assess the HDR-guided editing efficiency. For the latter, PCR and Sanger sequencing were used, as described above. To obtain homozygous cell clones, the edited cells were seeded at 0.5 cells/well in 100 μl, in 96-well plates. After >2-week expansion, individual cell clones were picked for genotyping. In the first step, the clones were screened by AgeI restriction digestion of the PCR amplicons since the C allele at rs8192678 creates an AgeI restriction site. Thus, a single band at 742 bp in the agarose gel indicates homozygous T/T and a single band at 632 bp indicates homozygous C/C genotype. All apparent homozygous or heterozygous clones screened by restriction digests were finally verified by Sanger sequencing using the genotyping forward primer as listed in ESM Table 1. This allowed us to detect the DNA sequence around ~600 bp upstream and ~100 bp downstream of the rs8192678-edited locus. Up to eight clonal populations of each homozygous genotype and six heterozygous clones were used in functional experiments.

### Standard PCR

For standard PCR amplification, a 25 μl reaction containing 1–10 ng genomic DNA, 2× Q5 PCR master mix (M0543L, New England Biolabs, USA), 0.4 µmol/l of appropriate forward and reverse primers and nuclease-free water were run on the BioRad C1000 Touch Thermal Cycler (BioRad, USA) using optimised cycling conditions with an initial step of 98°C for 30 s, followed by 30–35 cycles of 98°C for 10 s, 64–68°C (depending on the primer pairs) for 15 s and 72°C for 30 s to 1 min, with a final extension at 72°C for 2 min.

### Gene expression analysis

Total RNA was extracted from hWAs adipocytes using an RNeasy Plus kit (74136, Qiagen) according to the manufacturer’s instructions. RNA purity and concentration were measured with Nanodrop (Nanodrop, USA). cDNA was then synthesised using SuperScript IV VILO Master Mix (11756500, Thermo Fisher Scientific). The relative gene expressions were detected using the ViiA7 Real-Time PCR system (PE Applied Biosystems, USA) with pre-designed Taqman assays following the manufacturer’s instructions. The following pre-designed gene expression assays were ordered from Thermo Fisher Scientific: *PPARGC1A* (Hs00173304_m1), *PPARG* (Hs01115513_m1), *FABP4* (Hs01086177_m1), *CEBPA* (Hs00269972_s1), *SLC2A4* (Hs00168966_m1), *CEBPB* (Hs00942496_s1), *TOMM20* (Hs03276810_g1), *TFAM* (Hs01073348_g1), *PPARGC1B* (Hs00370186_m1), *LIPE* (Hs00193510_m1), *ABHD5* (Hs01104373_m1), *ACACB* (Hs01565914_m1), *CPT1B* (Hs00189258_m1), *CS* (Hs02574374_s1), *ADIPOQ* (Hs00977214_m1), *SCD* (Hs01682761_m1), *SREBF1* (Hs02561944_s1), *FASN* (Hs01005622_m1), *PNPLA2* (Hs00386101_m1), *LPL* (Hs00173425_m1), *MT-CO2* (Hs02596865_g1), *HPRT1* (Hs99999909_m1), *TBP* (Hs00427620_m1) and *RPL13A* (Hs03043885_g1). The geometric means of *TBP, HPRT1* and *RPL13A* housekeeping gene expression [20–22] were used to normalise the expression of genes of interest, and, unless indicated otherwise, the ΔΔC_t_ method was used to analyse the results.

### BODIPY and DAPI staining

For the lipid staining, the differentiated adipocytes were washed twice with PBS and fixed for 10–20 min with 4% (vol./vol.) buffered formalin at room temperature. The cells were then stained with BODIPY (2 mmol/l) solution (D3922, Thermo Fisher) for 15 min at room temperature, then washed five times with PBS. The cells were then incubated with DAPI (1 μg/ml) (D1306, Thermo Fisher) for 10 min (nuclear staining) and washed three times with PBS. The stained cells were visualised using a fluorescence microscope.

### Oil Red O and haematoxylin staining

Differentiated white adipocytes were stained with Oil Red O and haematoxylin as detailed in the ESM Methods.

### Immunofluorescence staining for perilipin-1

Differentiated white adipocytes were stained for perilipin-1 as detailed in the ESM Methods.

### Total triacylglycerol measurement in adipocytes

Triglyceride-Glo Assay kit (J3160, Promega, USA) was used to quantify total triacylglycerol content after adipogenic differentiation. Briefly, cells in a 24-well plate were incubated with 200 μl kit lysis buffer at 37°C for 30 min. After the reaction, 2.5 μl of each sample was diluted into 7.5 μl lysis buffer in the presence of lipase for 30 min and then diluted with 40 μl lysis buffer. For the glycerol detection, 10 μl of diluted samples was mixed with 10 μl of glycerol detection solution supplemented with reductase substrate and kinetic enhancer and transferred into a 384-well plate. After 1 h incubation at room temperature, the luminescence of each well was detected using the plate reader (CLARIOstar, BMG Labtech, Germany) and the triacylglycerol concentration of each sample was calculated using a standard curve generated from glycerol standards, normalised to total protein content obtained using BCA assays (23225, Thermo Fisher Scientific, Sweden).

### Seahorse bioenergetic profiling

To evaluate mitochondrial respiration, Seahorse XF (Seahorse Bioscience, USA) was used to measure oxygen consumption rate (OCR) in white adipocytes. Briefly, hWAs cells were seeded in a Seahorse 24-well plate and induced to differentiate using protocols described above. OCR was recorded continuously by sequentially adding 2 μmol/l oligomycin (EMD Chemicals, USA), 2 μmol/l FCCP and 5 μmol/l of the respiratory chain inhibitor rotenone at the indicated time points. After the measurement, the cell plate was then frozen at −80°C for at least 4 h, then the plate was dried and DNA was extracted with CyQUANT Cell Lysis Buffer (C7027, Thermo Fisher Scientific). Total DNA was then quantified using Quant-iT PicoGreen double-stranded DNA (dsDNA) assay kit (P7589, Thermo Fisher Scientific) against a lambda DNA-generated standard curve.

### Mitochondrial contents in rs8192678 C/C and T/T hWAs cells

To examine the effects of rs8192678 on mitochondrial biogenesis in differentiating white adipocytes, a relative amount of mitochondrial DNA (mtDNA) was quantified using a quantitative PCR (qPCR)-based method described before [23]. Briefly, total DNA was extracted and quantified using a QIAamp DNA Mini Kit (56304, Qiagen) at different days of differentiation (day 0, 3, 6 and 12). For qPCR, equal amounts of total DNA from each sample were mixed with SYBR Green Master Mix (A25742, Thermo Fisher Scientific) and with primers targeting mitochondrial and nuclear genes. The samples were then run on a ViiA7 Real-Time PCR system (PE Applied Biosystems, USA). The relative mtDNA content was calculated as ΔC_t_ (C_t_ of nuclear target – C_t_ of mitochondrial target).

### Cell proliferation assay

The cell proliferation reagent WST-1 (05015944001, Sigma-Aldrich, USA) was used to determine the proliferation rate of C/C and T/T cells. Cells were seeded at the density of 4000 cells per well in four 96-well plates, left to attach for 6 h, then the medium was supplemented with WST-1 reagent and incubated for 30 min (referred to as hour 0). Cellular proliferation was recorded by measuring absorbance at 450 nm (CLARIOstar, BMG Labtech, Germany). Similar measurements were done after 24, 48 and 72 h in the remaining three plates, and the relative cell numbers of each cell line were presented as relative to hour 0.

### Western blotting

After 12 days of differentiation, cells were washed twice with ice-cold PBS, lysed in 5% (wt/vol.) SDS for 10 min, and passed through Qiashredder (79656, Qiagen) for 5 min at 14,000 × *g*. The lysate was then transferred to a new tube and centrifugated for 15 min at 14,000 × *g*, the non-lipid aqueous phase was collected, and protein concentration was quantified using BCA assays (23225, Thermo Fisher Scientific). SDS-PAGE of 30 μg protein lysates was run on Any kD Mini-PROTEAN TGX Stain-Free Protein Gels (4568124, BioRad), and the proteins were transferred to PVDF membranes (1704156, BioRad). After blocking in 2–3% (wt/vol.) BSA solution for 1 h, the membranes were incubated with primary antibodies: anti-PGC-1α (ST-1202, Millipore, USA); oxidative phosphorylation (OXPHOS) complex antibodies (45-8099, Thermo Fisher Scientific); antibodies for fatty acid-binding protein 4 (FABP4), acetyl-CoA carboxylase (ACC), fatty acid synthase (FAS), CCAAT/enhancer-binding protein α (C/EBPα) and perilipin (12589, Cell Signaling Technology, USA); and GAPDH antibody (ab37168, Abcam, UK). The corresponding secondary antibodies were anti-mouse IgG (7076P2, Cell Signaling Technology) and anti-rabbit IgG (7074, Cell Signaling Technology). TBS with 0.1% (vol./vol.) Tween-20 was used for washing the membranes, and TBS with 1% (wt/vol.) BSA was used for antibody incubation. To visualise the blots, Clarity western electrochemiluminescence (ECL) substrate (1705060, BioRad) was added to the membrane and a charge-coupled device (CCD) camera and Image Lab software (BioRad, USA) were used to develop the images. Image J software [24] was used to quantify the protein bands.

### PGC-1α protein stability assay

After differentiation, the cells were incubated with 10 μmol/l cycloheximide for 0, 1 and 2 h to chase PGC-1α degradation. The cells were then washed with ice-cold PBS, lysed in 5% (wt/vol.) SDS buffer, and passed through Qiashredder (79656, Qiagen) for 5 min at 14,000 × *g*. The protein concentration of each sample was quantified and PGC-1α and GAPDH content was detected using western blot as described above.

### *PPARGC1A* mRNA stability assay

To study whether rs8192678 affects endogenous *PPARGC1A* mRNA stability, the cells were differentiated to adipocytes for 12 days. Next, the cells were washed twice with PBS and starved in DMEM medium with 2% (wt/vol.) fatty-acid-free BSA for 2 h. Subsequently, cells were incubated with or without actinomycin D (20 μg/ml) (SBR00013, Sigma-Aldrich) for 0.5 and 2 h. After the incubation, the cells were immediately harvested for total RNA extraction and cDNA synthesis, as described above. *PPARGC1A* mRNA was detected on a ViiA7 Real-Time PCR system (PE Applied Biosystems) using pre-designed Taqman assays following the manufacturer’s instructions, and the mRNA level after actinomycin D treatment was compared with that in untreated cells to investigate *PPARGC1A* mRNA stability.

### PPRE transcriptional activity luciferase reporter assays

Endogenous PGC-1α activity in C/C and T/T cells was measured using peroxisome proliferator-response element (PPRE) luciferase reporter assays, similarly to a method described previously [25]. Briefly, PPRE with the sequence AGGACAAAGGTCA, repeated three times in a sequence of 120 nucleotides, was synthesised as phosphorylated oligonucleotides (IDT), duplexed, and cloned into pGL4.23 vectors (Promega), upstream of a minimal promoter and the luciferase gene. For normalisation, *Renilla*–luciferase reporter vector pGL4.75 (Promega) was used. In each experiment, one to three C/C and T/T pre-adipocyte clones were electroporated with 95 ng pGL4.23 PPRE–luciferase reporter (PPRE-*luc2*) or pGL4.23 minimal promoter (control) and 5 ng *Renilla* pGL4.75 vectors (CMV-*Renilla*), followed by adipogenic differentiation induction 1 day post electroporation; the luciferase readout was measured 2 days post electroporation using a Dual-Glo Luciferase Assay System (Promega), following the manufacturer’s instructions. The luciferase signal was normalised to the *Renilla* signal, and the mean ratio PPRE/minimal promoter luciferase expression in each experiment was calculated.

### Lipogenesis assay

The method has been described previously [26]; briefly, at the end of the differentiation, the cells were incubated for 4.5 h with ^14^C-glucose labelling medium. The medium was made from glucose-free DMEM (Thermo Fisher Scientific, Sweden) with 2 mmol/l sodium pyruvate and 2 mmol/l l-glutamine, supplemented with 0.5 mmol/l d-glucose, 0.5 mmol/l acetate, 2% (wt/vol.) fatty-acid-free BSA and 7.4×10^4^ Bq/ml ^14^C-U-glucose (Perkin-Elmer, Sweden), with or without 1 μmol/l insulin. After the incubation, the cells were washed three times with cold PBS, and the lipids were extracted using Dole’s extraction medium. The radiolabelled lipids were quantified using liquid scintillation and normalised to total protein content measured using the BCA assay.

### Statistics

Randomisation and blinding were not carried out in this study, and no data were excluded from the results. For each assay, the number of biological replicates, SD and statistical significance are reported in the figure legends. Hypothesis tests for two groups were performed using two-tailed Student’s *t* test and multiple *t* tests, and the Mann–Whitney *U* test was applied when the normal distribution was not apparent. One-way ANOVA was used when comparing three or more groups. Two-way ANOVA was used to compare more than two groups with different conditions. A nominal *p* value of <0.05 was considered statistically significant. All analyses were undertaken using Prism Graphpad 9.0 software and Microsoft Excel 365.

## Results

### CRISPR-Cas9-mediated rs8192678 editing in hWAs pre-adipocyte cell lines

**Fig. 1:**
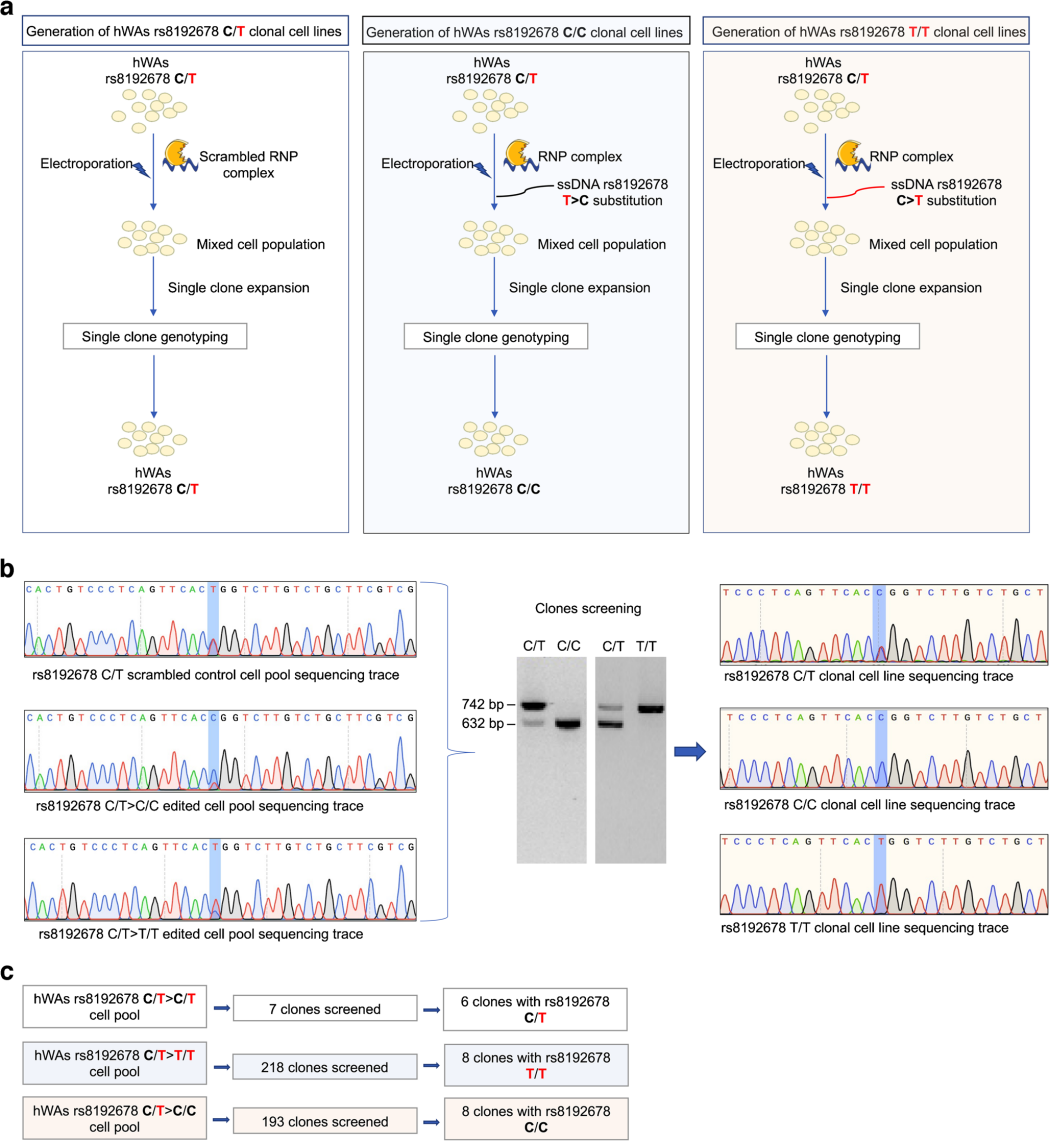
CRISPR-Cas9-mediated C-to-T and T-to-C allele substitution in hWAs pre-adipocyte cells. (**a**) Workflow for generation of rs8192678 C/T, C/C and T/T cell clones. (**b**) The left panel shows representative Sanger sequencing traces of C/T, C/C and T/T cell pools after CRISPR-Cas9-mediated editing. The middle panel shows AgeI restriction digests for clone screening. A single band at 742 bp indicates homozygous T/T clone and a single band at 632 bp indicates homozygous C/C clone. Two bands (742 bp and 632 bp) indicate a heterozygous C/T clone. The right panel shows representative Sanger sequencing traces of rs8192678 C/T, C/C and T/T clonal cell lines. (**c**) Diagram showing the number of screened clones and the number of edited clones after screening for each genotype

We used CRISPR-Cas9 to perform genomic allele editing in hWAs cells, to obtain all three genotypes at rs8192678 (C/T alleles). As shown in Fig. 1a, the hWAs genotype at rs8192678 is C/T; we therefore designed two sgRNAs (targeting the respective allele) and homologous donor templates to change C/T to either the C/C or T/T genotype. To obtain heterozygous cells, a scrambled RNP complex (mock) was applied. After single-cell cloning and clonal expansion, we generated homozygous and heterozygous populations that were identified by restriction digest screening and confirmed by Sanger sequencing (Fig. 1b). We eventually obtained eight C/C clonal populations after screening 193 clones from the T>C edited cell pool and eight T/T clonal populations after screening 218 clones from the C>T edited cell pool. For heterozygous C/T clones, we picked seven clones from the mock-edited cell pool, of which six clonal populations were growing well and confirmed to be C/T at rs8192678 (Fig. 1c). The edited clones were used in subsequent functional assays. To assess off-target effects of CRISPR-Cas9 we also applied the T7 endonuclease I (T7EI) assay to the top five predicted off-target sites after PCR amplification (predictions made using IDT CRISPR-Cas9 Design Checker tool: https://eu.idtdna.com/site/order/designtool/index/CRISPR_SEQUENCE, accessed on 25 Jan 2021). No off-target effects were detected (data not shown).

### The rs8192678 polymorphism regulates hWAs differentiation and lipid accumulation

**Fig. 2:**
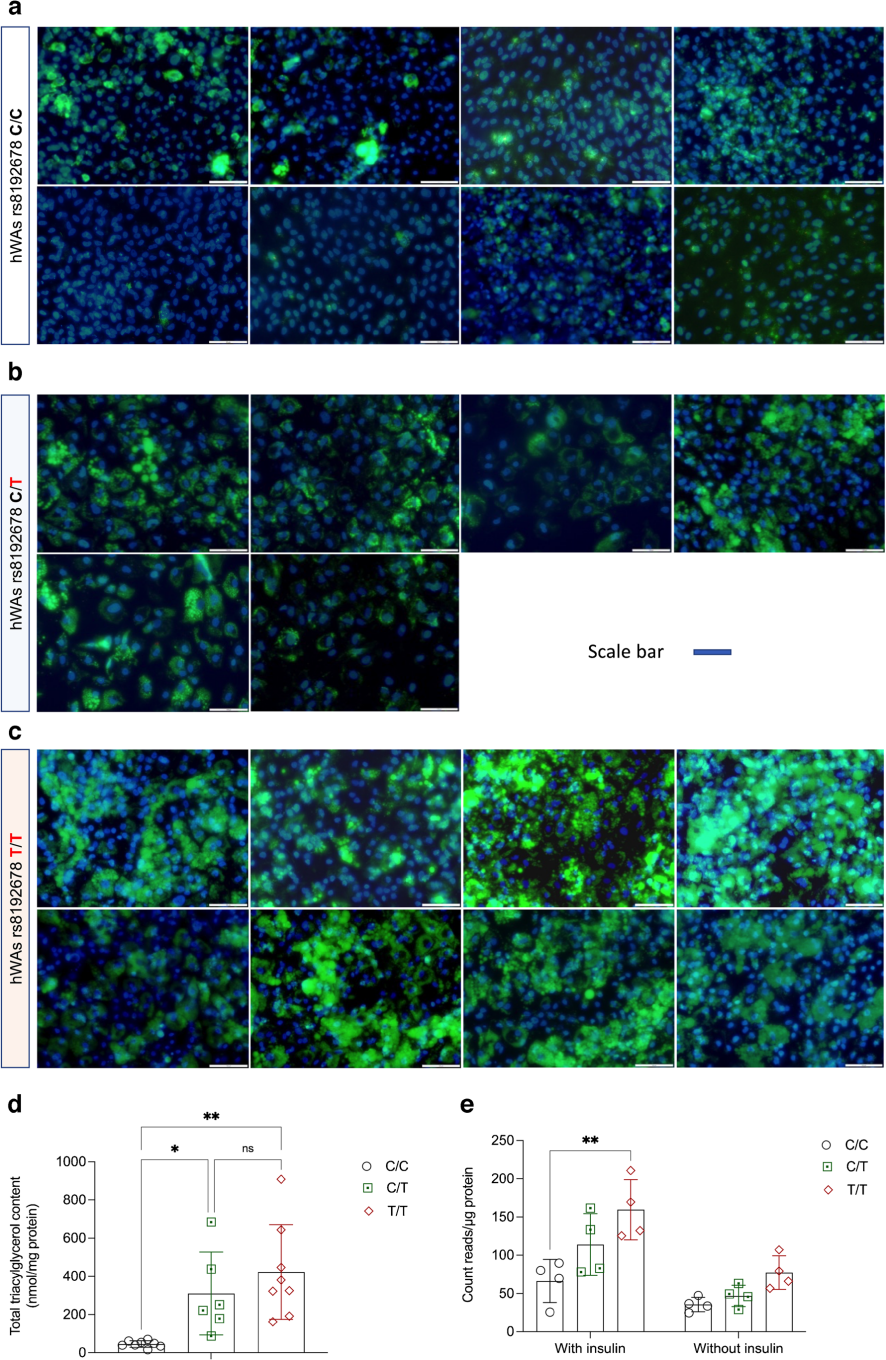
rs8192678 regulates adipocyte differentiation and lipid accumulation in hWAs clones. (**a–c**) BODIPY (green) and DAPI (blue) staining of hWAs C/C (**a**), C/T (**b**) and T/T (**c**) clones after differentiation induction (*n*=8 for C/C and T/T genotype, *n*=6 for C/T genotype); scale bar: 100 μm. (**d**) Biochemical quantification of total triacylglycerol normalised to total protein amount (*n*=8 for C/C and T/T genotype, *n*=6 for C/T genotype). Statistical analyses were performed using ordinary one-way ANOVA: *p<0.05, **p<0.01. Data are mean ± SD; ns, not significant. (**e**) Lipogenesis in C/C, C/T and T/T clones after differentiation induction. ^14^C-glucose incorporation into triacylglycerol was detected by liquid scintillation (count reads) and normalised to total protein. The measurements were done under two conditions: with or without insulin stimulation (*n*=4 for each genotype). Statistical analyses were performed using two-way ANOVA to compare the difference among all three genotypes: **p<0.01. Data are mean ± SD

**Fig. 3:**
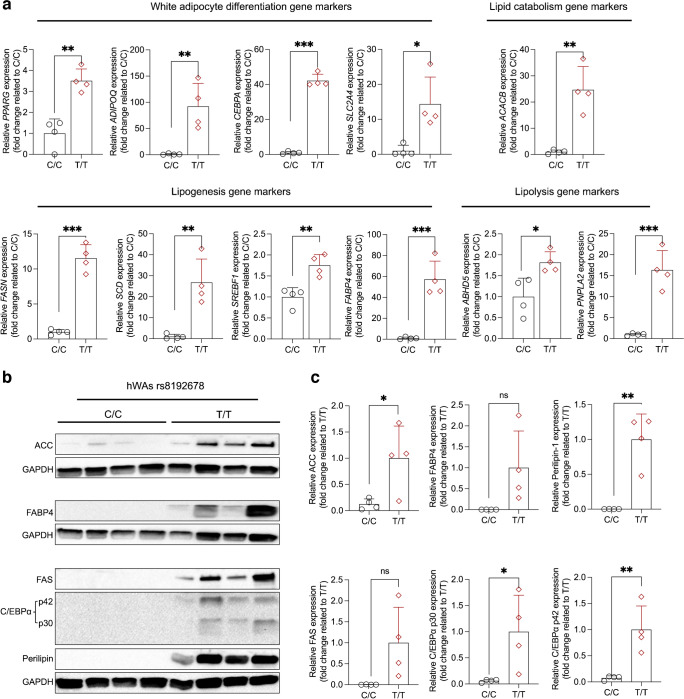
rs8192678 regulates adipocyte-related gene and protein expression in hWAs clones. (**a**) Relative expression of gene markers for adipocyte differentiation, lipid catabolism, lipogenesis and lipolysis in C/C and T/T homozygous clones after adipogenic differentiation (*n*=4 clonal populations per genotype). Statistical analyses were performed using two-tailed Student’s *t* test: *p<0.05, **p<0.01, ***p<0.001. Data are mean ± SD. (**b**) Immunoblots of adipogenesis markers of T/T and C/C cells (*n*=4 for each genotype). (**c**) Quantitative analysis of the relative band densities of the adipogenesis markers in (**b**). Statistical analysis was performed using two-tailed Student’s *t* test: *p<0.05, **p<0.01. Data are mean ± SD; ns, not significant

To evaluate the effects of rs8192678 allele editing on adipocytic phenotypes, we first differentiated the hWAs to mature adipocytes and morphologically assessed their lipid accumulation using BODIPY. We observed more BODIPY-positive cells in T/T and C/T vs C/C populations. We also biochemically quantified total triacylglycerol content and found it to be markedly higher in T/T and C/T cells (423 ± 247 nmol/mg protein and 310 ± 217 nmol/mg protein, respectively) than in C/C cells (46 ± 17 nmol/mg protein) (Fig. 2a–d, ESM Figs 1, 2). When assessing gene expression of adipogenic markers, we observed significantly higher transcript levels of *PPARG*, *ADIPOQ, CEBPA* and *SLC2A4*, and in T/T vs C/C cells (Fig. 3a). To further confirm the differentiation phenotype, we immunoblotted for several white adipose-specific proteins. Consistent with the mRNA data, the adipogenesis marker proteins ACC, C/EBPα and perilipin were significantly more highly expressed in T/T than in C/C cells; FABP4 and FAS were also more highly expressed in T/T that in C/C cells, although the difference did not reach the statistical significance threshold (*p*=0.063 for FABP4; *p*=0.057 for FAS) (Fig. 3b, c). To gain more insight into the early differentiation of T/T vs C/C adipocytes, we examined the expression of adipogenic markers at days 3 and 6 of the differentiation. As shown in ESM Results 1 and ESM Fig. 3, the expression of *PPARG* and *CEBPA* were higher in T/T cells already at day 3; the expression of *FABP4*, *CEBPA*, *ADIPOQ* and *FASN* became significantly higher at day 6. In another experimental setup, we examined how the absence of rosiglitazone affects the allele-edited hWAs differentiation, as rosiglitazone has previously been reported to interact with rs8192678 [27], and affect pre-adipocyte differentiation in vitro [28]. Although rosiglitazone only significantly improved *FABP4* expression in T/T but not in C/C cells, the expression of adipogenesis markers, the differentiation remained unequal between T/T and C/C adipocytes (ESM Results 2 and ESM Fig. 4). Taken together, these results suggest that the rs8192678 polymorphism affects white adipocyte differentiation in vitro, with the T allele conferring higher differentiation capacity.

### The rs8192678 polymorphism regulates hWAs lipogenesis

Given the striking effects of the rs8192678 polymorphism on triacylglycerol accumulation, to further explore the effects of rs8192678 on regulating lipid metabolism in hWAs adipocytes, we used ^14^C-labelled glucose to track de novo lipogenesis. As shown in Fig. 2e, at the basal condition, although the difference did not reach the nominal statistical significance threshold (*p*<0.05), T/T cells showed a higher lipogenesis (*p*=0.05) than C/C cells. When stimulated with insulin, lipogenesis increased in all three genotypes. Interestingly, the T allele showed an apparent additive dose-dependent effect on lipogenesis. Moreover, we evaluated lipid metabolism marker expression in T/T and C/C adipocytes. As shown in Fig. 3a, the T/T cells had significantly higher expression of the lipid catabolism marker gene *ACACB*, the lipogenesis marker genes *FASN, SCD, SREBF1* and *FABP4*, and the lipolysis marker genes *ABHD5* and *PNPLA2*. These data collectively illustrate the augmenting effect of the rs8192678 T allele on lipogenesis and lipid metabolism.

### The rs8192678 polymorphism affects mitochondrial respiration during hWAs differentiation

**Fig. 4:**
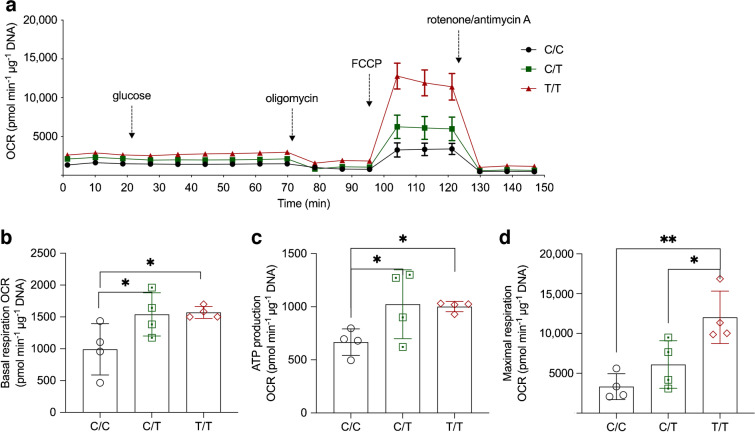
Cellular mitochondrial respiration in C/C, C/T and T/T hWAs clones. (**a**) Average OCR traces during basal respiration, and after addition of glucose, oligomycin, FCCP and rotenone/antimycin A. Data show mean ± SEM of *n*=4 for each genotype at each measurement point. (**b**) Extracted basal respiration OCR data from (**a**). (**c**) Extracted ATP-mediated respiration OCR data from (**a**) (ATP production OCR = OCR at basal condition – OCR after oligomycin treatment). (**d**) Extracted maximal respiration OCR data from (**a**) (maximal respiration OCR = OCR after FCCP treatment – OCR at basal level). Data show mean ± SD of *n*=4 for each genotype. Statistical analyses were performed using ordinary one-way ANOVA in (**b–d**): *p<0.05, **p<0.01

**Fig. 5:**
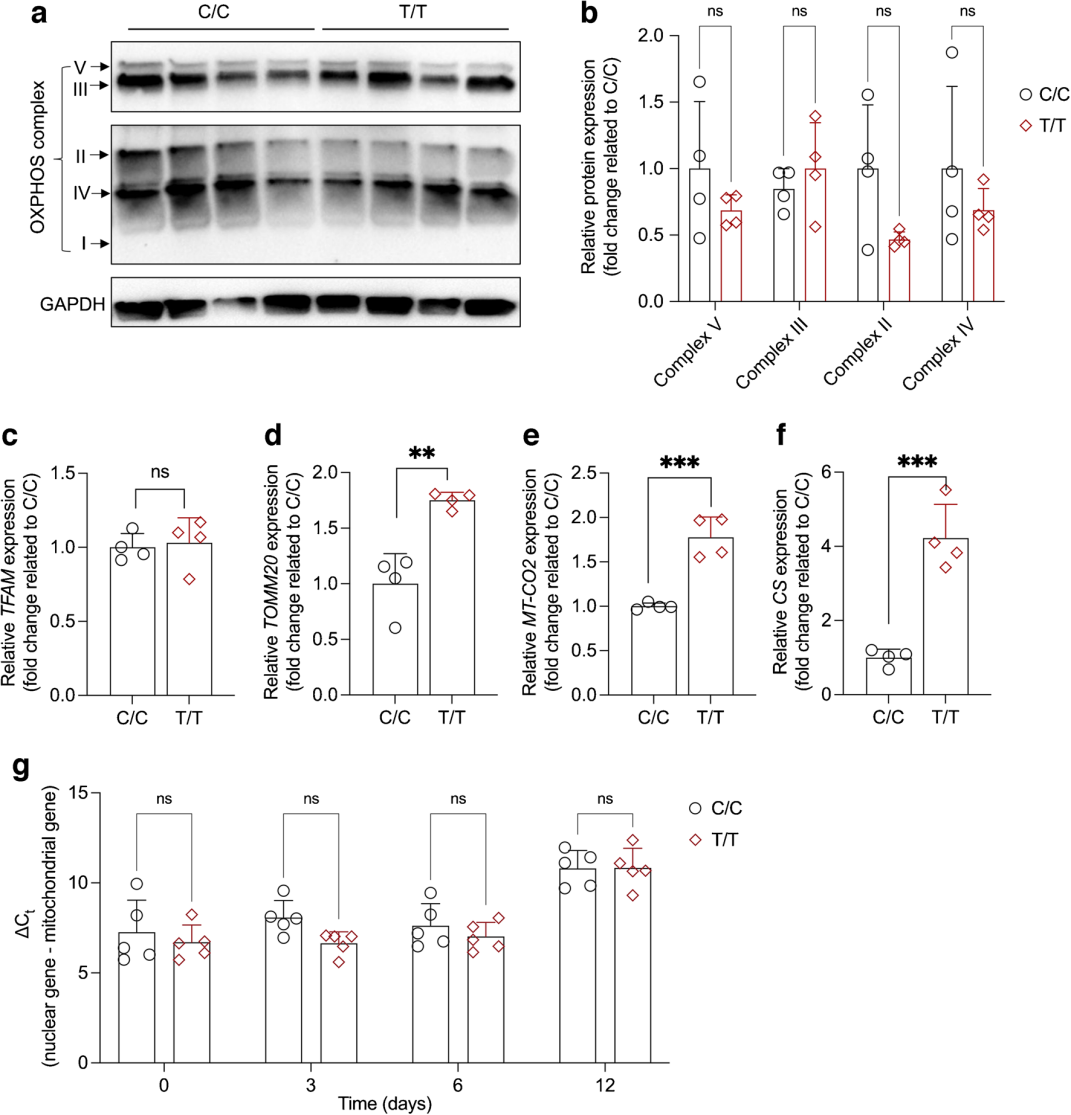
OXPHOS complex expression, mitochondrial content and mitochondrial gene marker expression in T/T and C/C adipocytes. (**a**) Immunoblots of mitochondrial respiration OXPHOS complexes in T/T and C/C cells (*n*=4 for each genotype). (**b**) Quantification of the relative band intensity fold change (related to C/C) in (**a**). Data show mean ± SD. Statistical analyses were performed using two-tailed multiple* t* test; ns, not significant. (**c**–**f**) Mitochondrial function gene markers expression (fold change related to C/C) in T/T and C/C cells after differentiation induction, *n*=4 for each genotype. Statistical analyses were performed using two-tailed Student’s *t* test: **p<0.01 ***p<0.001. Data show mean ± SD; ns, not significant. (**g**) Relative mitochondrial content in C/C and T/T hWAs on different days, *n*=5 for each genotype, data show mean ± SD; ns, not significant. Two-way ANOVA was used for comparing the means between T/T and C/C groups

Mitochondria are crucial for adipocyte differentiation [29] and PGC-1α appears to enhance mitochondrial biogenesis [30]. However, no published studies have investigated the role of rs8192678 in mitochondrial respiration during adipogenic differentiation. We hypothesised that our observed effects of rs8192678 allele editing on adipocyte differentiation were, at least in part, correlated with impaired mitochondrial function or content. To examine this, we measured mitochondrial OCR using a Seahorse extracellular flux analyser in C/C, C/T and T/T adipocytes. As shown in Fig. 4a, b, T/T and C/T adipocytes had higher OCR than C/C adipocytes at the basal respiration level. Furthermore, the addition of extra glucose to the assay medium did not increase the mitochondrial respiration in any of the three genotypes. Oligomycin, which blocks ATP synthase, resulted in less OCR decrease in C/C adipocytes, indicating lower ATP production OCR in C/C than in T/T and C/T cells (Fig. 4c). Adding the uncoupling agent FCCP, which collapses the proton gradient and disrupts the mitochondrial membrane potential, revealed that C/C and C/T adipocytes had significantly lower maximal respiration capacity than T/T adipocytes (Fig. 4d). To further explore the effects of the rs8192678 variant on mitochondrial function, we also examined the expression of OXPHOS complexes in T/T and C/C adipocytes. As shown in Fig. 5a, b, we found no significant differences in complex II, III, IV and V expression between T/T and C/C cells. However, as the human OXPHOS complex antibody only recognises one subunit of each complex, this result may not fully reflect the function of the mitochondrial respiration chain. Therefore, we also measured the mRNA expression of genes involved in mitochondrial function and found significantly higher transcript levels of *TOMM20*, *MT-CO2* and *CS* in T/T cells (Fig. 5c–f), suggesting higher mitochondrial function. Furthermore, we detected mtDNA content in differentiating C/C and T/T adipocytes, but did not find any significant differences at any time point (Fig. 5g). Interestingly, after 12 days of differentiation, mtDNA increased significantly but was still comparable in both T/T and C/C cells. Here, one should consider that the mitochondrial content in white adipocytes is low [31], which may hinder the detection of minor differences in mtDNA content. Collectively, our data indicate that the rs8192678 polymorphism influences white adipocyte differentiation through regulating mitochondrial oxidative respiration.

### The rs8192678 polymorphism does not affect hWAs pre-adipocyte proliferation

**Fig. 6:**
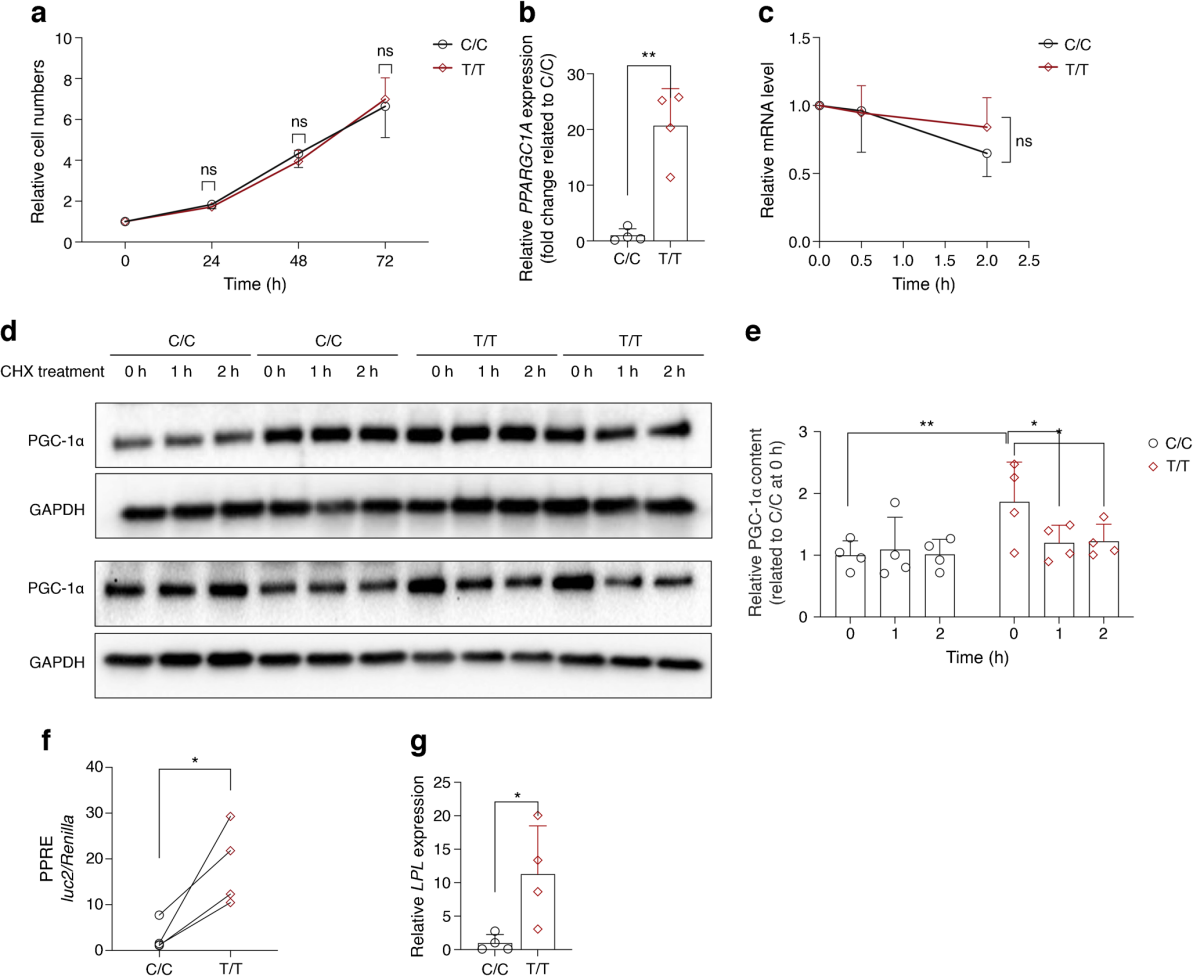
(**a**) Relative mean cell proliferation rate of C/C vs T/T pre-adipocytes. Data are presented as mean ± SD of *n*=5 clonal populations of each genotype. Multiple *t* test was used; ns, not significant. (**b**) Relative *PPARGC1A* mRNA expression (fold change related to C/C) in C/C and T/T cells after differentiation induction (*n*=4 for each genotype). Two-tailed Student’s *t* test was used: **p<0.01. Data are presented as mean ± SD. (**c**) Time course study of relative mRNA content in C/C and T/T cells after actinomycin D treatment. Data are presented as mean ± SD of *n*=4 for each genotype. Multiple *t* test was used for comparing the means between C/C and T/T groups at each time point; ns, not significant. (**d**) Immunoblots of PGC-1α in C/C and T/T cells with cycloheximide (CHX) treatment (*n*=4 clones for each genotype). (**e**) Quantification of the relative band intensity in (**d**). Data are presented as mean ± SD. Statistical analyses were performed using two-way ANOVA: *p<0.05, **p<0.01. (**f**) PPRE–luciferase reporter activity in C/C and T/T cells. The cells were transfected with PPRE–*luc2* and CMV*–Renilla* vectors, and PPAR activity was calculated as the ratio *luc2*/*Renilla*. Data are presented as mean ± SD of *n*=4 independent experiments. Two-tailed Student’s paired *t* test was used for comparing the means: *p<0.05. (**g**) PPARγ downstream gene target *LPL* expression in C/C and T/T adipocytes. Data are presented as mean ± SD of *n*=4 for each genotype. Two-tailed Student’s *t* test was used for comparing the means: *p<0.05

Given that the pre-adipocyte proliferation rate may affect adipogenesis in vitro [32], and given the observed blunted adipogenesis capacity in C/C cells, we examined the cellular proliferation rate in the rs8192678-edited hWAs. When measured over the course of 3 days after the initial seeding of the cells, no proliferation differences between T/T and C/C cells were apparent (Fig. 6a).

### The rs8192678 polymorphism regulates *PPARGC1A* mRNA expression and PGC-1α protein content and degradation in hWAs cells

To investigate potential molecular mechanisms underlying the effect of rs8192678 variation in adipogenesis, we determined *PPARGC1A* mRNA and PGC-1α protein expression and degradation. At the end of adipogenic differentiation, the *PPARGC1A* mRNA was higher in T/T than in C/C cells (Fig. 6b), but the endogenous *PPARGC1A* mRNA appeared not to degrade faster in C/C cells, when assayed using the transcription blocker actinomycin D (Fig. 6c).

With regard to PGC-1α protein content, previous studies in HepG2 and Ins-1 cells suggested altered PGC-1α stability for 482Ser vs 482Gly variants [15, 16]; these studies, however, utilised ectopically overexpressed PGC-1α, which may have confounded the readout owing to cellular processes altered by extreme *PPARGC1A* overexpression. Here, we treated the edited T/T and C/C human white adipocytes with cycloheximide (protein translation blocker) to chase PGC-1α protein degradation in T/T and C/C cells. As shown in Fig. 6d, e, in T/T adipocytes, the PGC-1α protein contents decreased rapidly after 1 h cycloheximide treatment (*p*=0.03 when compared to 0 h), and remained at low level after 2 h treatment (*p*=0.04 when compared to 0 h). Changes were not significant between 1 h and 2 h (*p*=0.9). In C/C adipocytes, on the other hand, the PGC-1α content remained largely unchanged at all time points. This may reflect impaired degradation of the 482Gly PGC-1α variant through aggregation. This interpretation is coherent with cycloheximide stopping the synthesis of protein-degrading enzymes [33–35], and not completely stopping protein translation [36, 37]. The data in Fig. 6d, e also shows that the steady state (0 h) content of PGC-1α in C/C cells is significantly lower than in T/T cells, which is consistent with the mRNA expression differences shown in Fig. 6b. Taken together, these data suggest rs8192678 482Gly (C allele) confers lower expression of *PPARGC1A* mRNA and PGC-1α protein. Furthermore, the rs8192678 polymorphism does not affect *PPARGC1A* mRNA stability but can affect PGC-1α protein degradation.

### The rs8192678 polymorphism affects PPARγ transcriptional activity

Peroxisome proliferator-activated receptor γ (PPARγ) has been identified as a master regulator of adipocyte differentiation, partly because of its regulatory role in metabolism-related gene expression [38]. Since the transcriptional activity of PPARγ can be regulated by its coactivator PGC-1α [39], and because we observed a higher content of 482Ser vs 482Gly PGC-1α, it was also likely that the PPARγ transcriptional activity was affected in our rs8192678-edited cells. To test this hypothesis, we transfected PPRE–luciferase reporter plasmid into C/C and T/T cells, which allowed us to evaluate their endogenous PPARγ transcriptional activity. Here, we observed that T/T cells had higher PPRE–luciferase expression (Fig. 6f). To further validate these findings, we also quantified the expression of the previously described [40] PPARγ downstream targets *LPL*, *FABP4*, *CEBPA*, *ADIPOQ* and *FASN*. As shown in Figs 3a and 6g, all the targets are expressed at significantly higher levels in T/T than in C/C cells. Collectively, these data show that T/T cells have higher PPARγ transcriptional activity.

## Discussion

Several epidemiological studies have linked *PPARGC1A* variation at rs8192678 with obesity and other cardiometabolic diseases [8, 41–48]. The T allele (482Ser) has been associated with lower NEFA levels, smaller adipocyte size, and higher lipid oxidation in Native Americans [49], but with reduced NEFA clearance after oral glucose challenge in people of European ancestry [50]. Observational studies have also linked the T allele or decreased *PPARGC1A* expression with insulin resistance or excess adiposity in adults and children of varying ethnicities [39, 42–44, 51]. Similar phenotypes have been reported in fat-fed adipose-specific *Ppargc1a*-deficient mice [52]. Collectively, these data suggest that the rs8192678 T allele may affect adipocyte differentiation and lipid metabolism in vivo.

rs8192678 is a missense variant and the only common polymorphism in its haploblock. To determine whether it is likely to play a functional role in human adipocytes, we used CRISPR-Cas9 to generate isogenic human white pre-adipocyte (hWAs) cell lines homozygous or heterozygous for the rs8192678 C and T alleles. After adipogenic differentiation, the C allele (482Gly) decreased triacylglycerol content, lipogenesis and expression of adipogenic markers, as well as lipid metabolism markers (Figs 2, 3). The T allele, conversely, appeared to improve lipogenesis and triacylglycerol accumulation in an additive dose-dependent manner (Fig. 2). The T allele also conferred higher PPARγ activity (Fig. 6f), which is consistent with a previous study that used ectopic overexpression of the 482Ser PGC-1α [53]. However, the increased PPARγ activity we found in T/T cells might simply be due to the increased PPARγ and PGC-1α expression caused by the higher adipocyte differentiation efficiency. We also found that the T/T cells had higher mitochondrial activity (Fig. 4), probably in part owing to improved adipogenic differentiation [7]. The improved mitochondrial function could explain why the T allele carriers appear to benefit more from weight loss interventions, such as energy-restricted diets [54], bariatric surgery [55] and acarbose treatment [12].

While the mechanism for the higher differentiation capacity in the rs8192678 T/T cells could well be explained by the higher PGC-1α content modulating PPARγ activity, it may also be explained by a faster PGC-1α protein turnover rate imposed by the Gly-to-Ser substitution (Fig. 6d, e). The faster degradation rate of the 482Ser PGC-1α protein may also explain the previously reported lower *PPARGC1A* mRNA in pancreas and muscle in T allele carriers [13, 14], as PGC-1α expression is to a degree self-regulating [56].

Judging from this and previous studies, a fast PGC-1α turnover rate, and not merely its high content, appears critical for basal cellular function and differentiation [57]. Being a key transcriptional coactivator in energy metabolism, PGC-1α requires stringent regulation to quickly respond to shifting metabolic demands and to reduce interference with shifting metabolic pathways (e.g. adipogenesis vs lipolysis) [38]. The half-life of PGC-1α is therefore short [58] and tightly controlled by 20S– and ubiquitin–proteasome-mediated degradation [59, 60]. In this context, both PGC-1α content and turnover rate imposed by the rs8192678 missense mutation appear to have profound consequences for adipogenic differentiation and metabolism.

Our findings further expand on the previously reported role of *PPARGC1A* in adipocyte differentiation. *PPARGC1A* knockdown in human mesenchymal stem cells affects differentiation to brown adipocytes [61], which is coherent with an increased *PPARGC1A* expression in ex vivo differentiating human subcutaneous adipocytes [62]. Interestingly, *Ppargc1a*-deficient mice have lower body fat mass both under normal feeding and high-fat feeding conditions [63], yet both *Ppargc1a*-deficient and wild-type primary white pre-adipocyte cells differentiate equally well and accumulate similar amounts of lipids [52]. Similarly, *Ppargc1a* deficiency does not affect the differentiation of mouse pre-adipocytes to brown adipocytes, although it alters thermogenic gene expression [64]. Collectively, these data and those from our study indicate a species- and tissue-dependent role of *PPARGC1A* in adipocyte differentiation. These data also suggest that the outcomes of experiments based on genomic editing of single nucleotide variants may not be readily extrapolated from results obtained in gene knockout experiments. This is worth considering in studies seeking to determine the functional role of other human genetic polymorphisms.

### Limitations of the study

There are several limitations of this study. It detected disparate effects of the two rs8192678 alleles on adipogenic differentiation *in vitro*. Because we could not perform well-controlled gene-by-environment interaction experiments in similarly differentiated cells with similar triacylglycerol content, we were unable to validate some of the epidemiological findings, e.g. insulin sensitivity, experimentally. Another limitation of our study, mainly resource-related, is the use of CRISPR-Cas9 to edit just one cell type, from a single genetic background. Nevertheless, regardless of these limitations, the effect size on several variables appears meaningful.

### Conclusions

In summary, although many studies have linked rs8192678 to metabolic disorders, the conclusion on whether the rs8192678 T allele is detrimental or protective may depend on the environmental context, the tissue-specific functions of the allele, and perhaps even the stage of disease progression. For example, T allele carriers have increased risk for adiposity and type 2 diabetes [43–45, 65], but also respond better to certain lifestyle interventions [12, 54, 55]. Mechanistically, the rs8192678 T allele encodes 482Ser, which may create a phosphorylation site [16] and may result in PGC-1α 482Ser being more sensitive to environmental cues in adipose cells.

Linking our current experimental data to the observational findings is difficult as our cell model only addresses the white adipocyte function in in vitro experiments, as opposed to epidemiological data that can be collected from individuals with extensive disease progression. It is surprising that the T allele confers improved adipocyte function in our experiments, as it is also associated with metabolic disorders. Here, one has to consider that whole-body metabolic dysregulation can be influenced by the T allele action in other tissues, including liver, muscle and brain. Our experimental data, showing the T allele-improved adipogenic differentiation capacity, appears, at least in part, coherent with T allele carriers having higher body fat mass and BMI, and excessive weight gain [42–46], although the long-term in vivo effects of the allele on other variables (e.g. insulin sensitivity) remain to be investigated. Taken together, our investigation suggests that the rs8192678 T allele enhances white adipocyte differentiation and mitochondrial function. More data on the function of this allele in other tissues are needed to gain fuller understanding on how rs8192678 affects whole-body metabolism.

## Supplementary Information

Below is the link to the electronic supplementary material.

Supplementary file1 (PDF 748 KB)

## Data Availability

All data generated in our current study are available upon reasonable request.

## Abbreviations

ACC: Acetyl-CoA carboxylase
C/EBPα: CCAAT/enhancer-binding protein alpha
FABP4: Fatty acid-binding protein 4
FAS: Fatty acid synthase
hWAs: Human pre-adipocyte white adipose tissue (cell line)
mtDNA: Mitochondrial DNA
OCR: Oxygen consumption rate
OXPHOS: Oxidative phosphorylation
PGC-1α: Peroxisome proliferator-activated receptor γ coactivator 1-α
PPARγ: Peroxisome proliferator-activated receptor γ
PPRE: Peroxisome proliferator-response element
qPCR: Quantitative PCR
RNP: Ribonucleoprotein
sgRNA: Single guide RNA
ssDNA: Single-stranded DNA
WAT: White adipose tissue
