# Supplementary material for: Engineered allele substitution at PPARGC1A rs8192678 alters human white adipocyte differentiation, lipogenesis, and PGC-1α content and turnover
Source: Diabetologia. 2023 May 12;66(7):1289–305. doi: 10.1007/s00125-023-05915-6 (PMC10244287; doi:10.1007/s00125-023-05915-6)
Supplement: Supplementary file 1 — Supplementary file1 (PDF 748 KB) [file 125_2023_5915_MOESM1_ESM.pdf]

ESM Methods

Commented [MOU1]: Note we have removed numeric labels (1,2) for the different paragraphs in ESM Methods. Please feel free to reinstate them though, if you deem them necessary.

Oil Red O and hematoxylin staining

The differentiated white adipocytes on day 12 and day 28 were washed twice with PBS and fixed for 10-20 minutes with 4% buffered formalin at room temperature. The cells were stained with Oil Red O solution for 30 minutes at room temperature, then washed 5 times with distilled water. The cells were then incubated with hematoxylin solution (MHS32, Sigma-Aldrich) for one minute (nuclear staining) and washed three times with water. The stained cells were visualized using light microscopy, as shown in **ESM Fig. 1**.

Immunofluorescence staining for perilipin-1 in differentiated adipocytes

The differentiated white adipocytes on day 28 were washed twice and fixed with 4% paraformaldehyde for 15 minutes at room temperature prior to immunofluorescence staining. The cells were then incubated in blocking buffer (5% Donkey normal serum in PBS with 0.3% Triton X-100) for 1 hour and followed by perilipin-1 primary antibody (#9349, Cell signaling technology) incubation overnight at 4°C. After washing with PBS three times, cells were incubated with donkey anti-rabbit secondary antibody (A-31572 Alexa Fluor™ 555, Thermofisher Scientific) for 1 hour and perilipin-1 (red fluorescence) was then visualized by a confocal microscope, as shown in **ESM Fig. 2**.

ESM Results 1

Adipocyte differentiation markers expression were different between C/C and T/T adipocytes on differentiation day 3 and 6

To gain a better understanding of the unequal differentiation efficiency between C/C and T/T adipocytes, we quantified adipocyte gene markers at early days of the differentiation process.

As shown in **ESM Fig. 3**, on day 3, the expression of *PPARG* and *CEBPA* were significantly higher in T/T than C/C cells, while other markers *PPARGC1A*, *PPARG*, *ADIPOQ*, *CEBPB*, *FABP4* and *FASN* were not statistical different between T/T and C/C cells. On day 6, *FABP4*, *PPARG*, *CEBPB*, *ADIPOQ*, *FASN* and *CEBPA* expression became significantly higher in T/T than C/C cells, while *CEBPB and SREBF1* levels were comparable. The *PPARGC1A* expression was considerably higher in T/T cells, although due to large intra-group variance it yields no statistical significance (p = 0.07). These data indicate the adipocyte differentiation program is affected by rs8192678 already during early adipogenic differentiation.

ESM Results 2

Rosiglitazone did not markedly change the adipogenic differentiation of C/C versus T/T cells

To investigate if rosiglitazone affects C/C and T/T preadipocyte differentiation, we differentiated the cells with or without rosiglitazone in the differentiation medium for the first 6 days, then quantified the adipocyte differentiation marker gene expression after 12 days. As shown in **ESM Fig. 4**, in the absence of rosiglitazone in the differentiation medium, T/T cells showed significantly higher expression of *PPARGC1A*, *PPARG*, *SREBF1*, *ADIPOQ* and *FABP4*, indicating T/T cells have higher adipocytes differentiation capacity than C/C cells.

ESM Table 1 DNA sequences of genotyping primers, and CRISPR/Cas9 sgRNAs and donor templates used in genotyping and allele editing of rs8192678

Genotyping primer Forward sequence	5´- AGGGCAGCTCTCCAGGTAAT - 3´
Genotyping primer Reverse sequence	5´- CCTTGCAGCACAAGAAAACA - 3´
rs8192678 (C-to-T) sgRNA spacer sequence	5´- GACGACGAAGCAGACAAGAC - 3´
rs8192678 (T-to-C) sgRNA spacer sequence	5´- CAGACAAGACCAGTGAACTG - 3´
Single strand oligo DNA donor template for C>T allele switch	5´ - ACTTCGGTCATCCCAGTCAAGCTGTTTTTGACGACGAAGCAGACAAGACCAGTGAACTGAGGGACAGTGATTTCAGTAATGAACAATTCTCCAAACTACC - 3´
Single strand oligo DNA donor template for T>C allele switch	5´- ACTTCGGTCATCCCAGTCAAGCTGTTTTTGACGACGAAGCAGACAAGACCGGTGAACTGAGGGACAGTGATTTCAGTAATGAACAATTCTCCAAACTACC - 3´

ESM Fig. 1

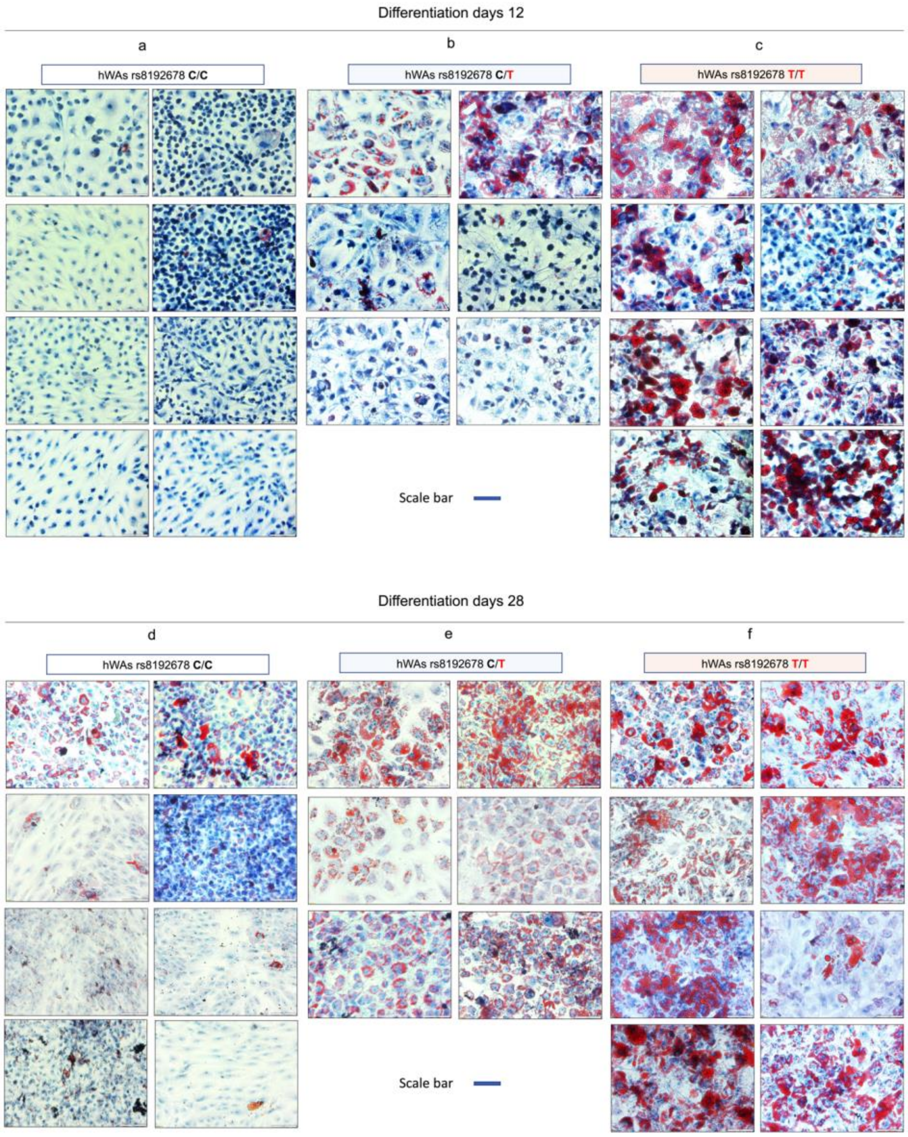

ESM Fig. 1 rs8192678 regulates adipocyte differentiation and lipid accumulation in hWAs clones. **a-c.** Oil Red O and hematoxylin staining of hWAs C/C, C/T and T/T clones after 12

days of differentiation induction (*n*=8 for C/C and T/T genotype, *n*=6 for C/T genotype). Scale bar: 100 µm. **d-f.** Oil Red O and hematoxylin staining of hWAs C/C, C/T and T/T clones after 28 days of differentiation induction (*n*=8 for C/C and T/T genotype, *n*=6 for C/T genotype). Scale bar: 100 µm.

ESM Fig. 2

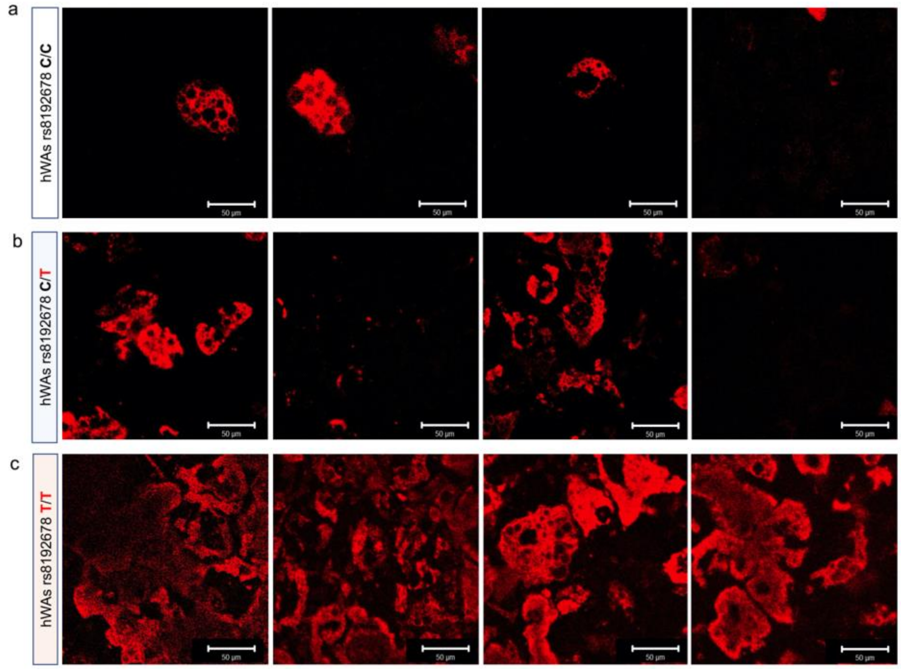

ESM Fig. 2. a. Perilipin-1 staining in rs8192678 C/C cells (*n*=4). **b.** Perilipin-1 staining in rs8192678 C/T cells (*n*=4). **c.** Perilipin-1 staining in rs8192678 T/T cells (*n*=4).

ESM Fig. 3

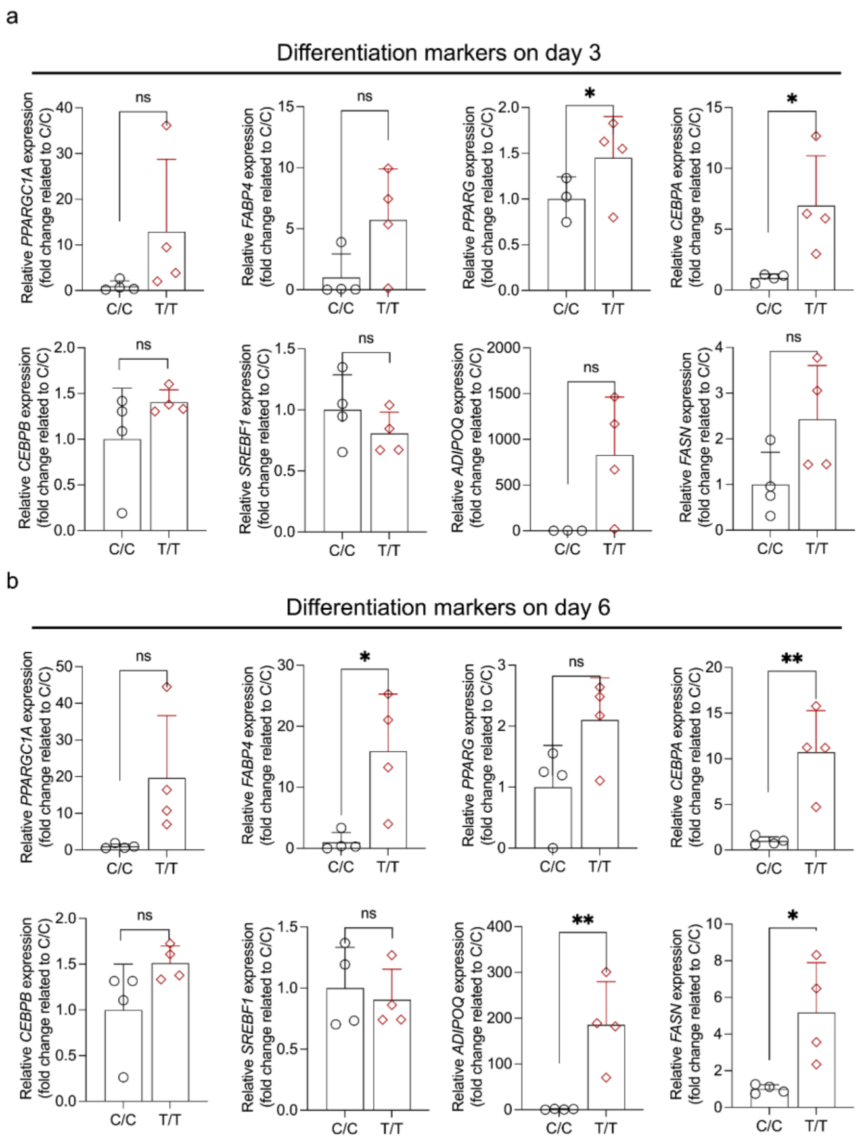

ESM Fig. 3 Differentiation marker gene expression in T/T and C/C cells on differentiation day 3 and day 6, *n*=4 for each genotype in all figures. Statistical analyses were performed using

two-tailed Student´s t test. Data show mean ± SD, *p<0.05, **p<0.01 was used to present statistical significance, `ns` represents no statistical significance.

ESM Fig. 4

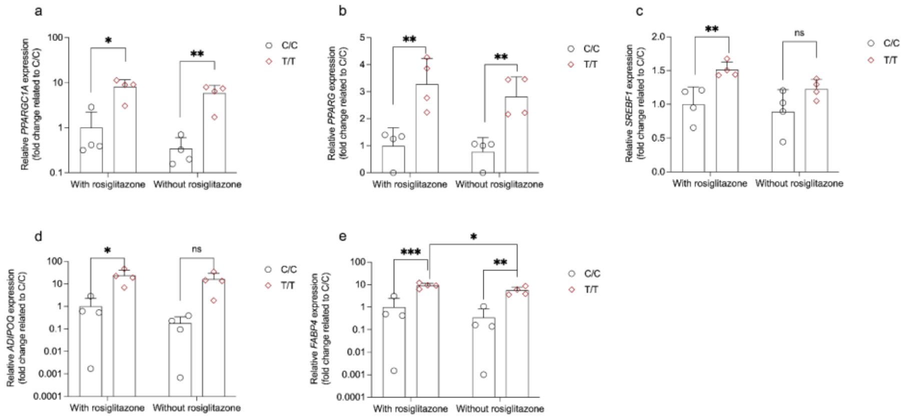

ESM Fig. 4 Relative adipocyte gene marker expression in T/T and C/C cells after the differentiation with and without rosiglitazone. *n*=4 clonal populations per genotype were used in the experiment, and the statistical analyses were performed using two-way ANOVA. Data show mean ± SD, *p<0.05, **p<0.01, ***p<0.001 was used to present statistical significance, `ns` represents no statistical significance.
